# Contrast Enhancement without Transient Map Expansion for Species-Specific Vocalizations in Core Auditory Cortex during Learning

**DOI:** 10.1523/ENEURO.0318-16.2016

**Published:** 2016-11-30

**Authors:** Kathryn N. Shepard, Kelly K. Chong, Robert C. Liu

**Affiliations:** 1 Graduate Program in Neuroscience, Emory University, Atlanta, Georgia 30322; 2 Department of Biology, Emory University, Atlanta, Georgia 30322; 3 Wallace H. Coulter Department of Biomedical Engineering, Georgia Institute of Technology, Atlanta, Georgia 30332; 4 Center for Translational Social Neuroscience, Emory University, Atlanta, Georgia, 30322

**Keywords:** auditory learning, lateral inhibition, map plasticity, maternal behavior, tonotopic map, ultrasonic vocalization

## Abstract

Tonotopic map plasticity in the adult auditory cortex (AC) is a well established and oft-cited measure of auditory associative learning in classical conditioning paradigms. However, its necessity as an enduring memory trace has been debated, especially given a recent finding that the areal expansion of core AC tuned to a newly relevant frequency range may arise only transiently to support auditory learning. This has been reinforced by an ethological paradigm showing that map expansion is not observed for ultrasonic vocalizations (USVs) or for ultrasound frequencies in postweaning dams for whom USVs emitted by pups acquire behavioral relevance. However, whether transient expansion occurs during maternal experience is not known, and could help to reveal the generality of cortical map expansion as a correlate for auditory learning. We thus mapped the auditory cortices of maternal mice at postnatal time points surrounding the peak in pup USV emission, but found no evidence of frequency map expansion for the behaviorally relevant high ultrasound range in AC. Instead, regions tuned to low frequencies outside of the ultrasound range show progressively greater suppression of activity in response to the playback of ultrasounds or pup USVs for maternally experienced animals assessed at their pups’ postnatal day 9 (P9) to P10, or postweaning. This provides new evidence for a lateral-band suppression mechanism elicited by behaviorally meaningful USVs, likely enhancing their population-level signal-to-noise ratio. These results demonstrate that tonotopic map enlargement has limits as a construct for conceptualizing how experience leaves neural memory traces within sensory cortex in the context of ethological auditory learning.

## Visual Abstract

**Figure:**
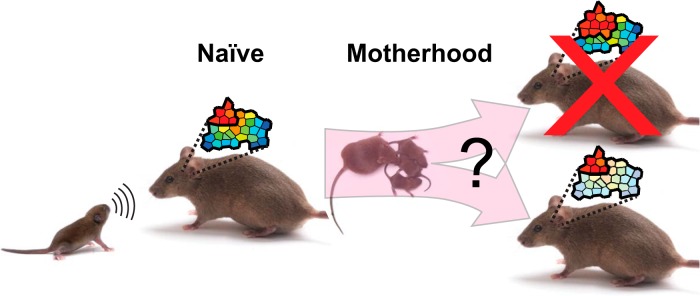

## Significance Statement

A commonly held impression in neuroscience is that learning the behavioral meaning of a stimulus expands the sensory cortical territory representing it, at least transiently if not persistently. Here we investigated whether such expansion occurs in a natural sound-learning paradigm wherein mouse mothers come to recognize the importance of pup ultrasonic vocalizations over the course of pup care. Contrary to expectation, we found no evidence for transient or sustained expansion in the representational area of these calls, but instead observed an enhancement in the contrast between how neurons tuned to and away from ultrasonic frequencies respond to those calls. This work has relevance for understanding the neural correlates of learning in evolved species-specific communication systems.

## Introduction

A hallmark of the organization of primary sensory cortices is the topographic gradient in the representation of the sensory epithelium, whether this is retinotopic (Talbot and Marshall, 1941; Daniel and Whitteridge, 1961), somatotopic (Adrian, 1941), or cochleotopic (i.e., tonotopic) (Woolsey and Walzl, 1942; Merzenich et al., 1975). Such topographical representations of stimulus “location” can be shaped by sensory experience, a phenomenon known as map plasticity, which has been well explored in the auditory domain (Recanzone et al., 1993; Pienkowski and Eggermont, 2011; Schreiner and Polley, 2014). Passive, prolonged pure tone exposure during a critical period of development can robustly expand the area within core auditory cortex (AC) with characteristic frequencies attuned to that sound (Zhang et al., 2001; De Villers-Sidani et al., 2007; Barkat et al., 2011), which can then bias adult perceptual discrimination of nearby frequencies (Han et al., 2007). In adulthood, map expansion has been observed in various associative tone-learning paradigms (e.g., classical and operant conditioning), which has led to the hypothesis that map expansion itself is a neural engram of the auditory memory for a behaviorally relevant frequency (Weinberger, 2004; Rutkowski and Weinberger, 2005; Bieszczad and Weinberger, 2010b). However, other studies have not consistently found expansion (Brown et al., 2004; Berlau and Weinberger, 2008), potentially because it can be sensitive to experimental factors like motivation or learning strategy (Bieszczad and Weinberger, 2010a). Moreover, behavioral performance in a learned auditory task for a trained frequency can remain high even if AC map expansion for that frequency has faded, suggesting that map expansion may only be a transient phase in auditory learning (Reed et al., 2011).

Given the debate over the role of map plasticity in auditory learning and memory, investigating such plasticity in a more ethological context would inform whether this correlate is present in learning tasks for which evolutionary pressures have likely shaped the auditory system. In fact, in an earlier study of auditory plasticity for communication sounds, tonotopic plasticity at the map level was not observed after those sounds gained behavioral relevance, despite plasticity on a smaller size scale observed at the single-neuron level (Cohen et al., 2011; Marlin et al., 2015; Shepard et al., 2015b) and local population level (Galindo-Leon et al., 2009; Rothschild et al., 2013). Specifically, mouse pups displaced from their nest emit ultrasonic vocalizations (USVs), which maternal mice, but not pup-naive virgins, preferentially approach in search of lost pups. Since pup USVs resemble pure tones in their flat-frequency trajectories and lack of harmonics, learning the behavioral relevance of these calls through their pairing with pups was hypothesized to yield AC map expansion for USVs or ultrasound tones (>40 kHz). Though this was not observed after natural maternal experience, the hypothesis that transient map expansion occurs during experience to support memory formation has not been tested.

To investigate this hypothesis, we electrophysiologically mapped the auditory cortices of pup-naive females and three groups of maternal mice at different time points throughout pup rearing. Time points before and after peak pup USV production, which occurs at postnatal day 7 (P7), were chosen to maximize the likelihood of capturing a transient peak in map plasticity in lactating dams. We found that the areal representation of USVs or ultrasonic best frequencies (BFs) across the tonotopic map remained stable across all maternal time points, indicating that map expansion is not among the plasticity events that occur to support maternal sensitization to pup USVs. However, we did find plasticity in the magnitude of the USV-evoked neural population response across different portions of core AC. Specifically, we observed a suppression of ultrasound-evoked responses in regions tuned to frequencies outside the ultrasound range, without a concomitant change in regions tuned to ultrasonic frequencies, a population-level contrast enhancement mechanism that may facilitate the detection of pup USVs.

## Materials and Methods

All procedures were approved by the Emory University Institutional Animal Care and Use Committee. Electrophysiological recordings of pure tone responses were taken across the ACs of pup-naive virgin females (*n* = 8) and separate groups of dams at different postnatal time points (P3 to P4 after parturition, *n* = 7; P9 to P10 after parturition, *n* = 8; and at pup weaning, approximately 21 d after parturition, *n* = 8). All subjects were *CBA/CaJ* mice (RRID: IMSR_JAX:000654) that were 11–17 weeks of age at the time of recording. All maternal animals had natural home-cage experience with their own pups, including with their USVs, prior to electrophysiological recording (duration of experience depended on which maternal group an animal was assigned to).

### Surgical procedures

On the morning of recording, dams were removed from their litters at the time of anesthetic induction while pups were killed with an overdose of isoflurane. Animals undergoing electrophysiological mapping were anesthetically induced with a ketamine (100 mg/kg) and xylazine (5 mg/kg) cocktail (6:1) delivered intraperitoneally. Maintenance doses of ketamine (30 mg/kg) and xylazine (1 mg/kg) were delivered via an intraperitoneal cannula when a toe pinch reflex was observed (usually every 20–30 min). The heads of animals were secured in a stereotax with bite bar (Model 900, David Kopf Instruments), and 2% oxygen was delivered through tubing attached to a sliding nose clamp. Fur on top of the head was trimmed, and an incision was made down the midline of the scalp. Schwartz vessel clips (World Precision Instruments) were applied along the edges of the incision to further open the wound and reveal the underlying skull. A periosteal elevator was used to detach the left temporal muscle from the skull until the zygomatic arch became visible. This portion of the temporal muscle was then cut away to facilitate access to the skull directly overlying the temporal cortex.

Craniotomy boundaries were labeled with a nontoxic, water-resistant marker. The rostral boundary was placed at 30% of the distance between bregma and lambda, the caudal boundary was placed at 90% of the distance between bregma and lambda, the ventral boundary was placed immediately above the zygomatic arch, and the dorsal boundary was placed ∼1 mm dorsal to the ridge separating the temporal and dorsal surfaces of the skull. After these boundaries were delineated, an inverted flat-head machine screw (0.19 inch head diameter × 0.47 inch length) was placed immediately behind bregma and secured in place with dental cement (MaxCem; Kerr); this screw served as a headpost to maintain head position throughout the recording. Another flat-head machine screw (0.054 inch head diameter × 0.09 inch length) was driven into the skull overlying the frontal lobe to serve as an electrical ground. Prior to the craniotomy, animals were removed from the stereotax, and their headposts were anchored in a mount. The craniotomy was performed with either a hand-held Dremel tool with #1/4 carbide bur and/or a scalpel with #11 blade. Upon completion of the craniotomy, silicon oil was applied to the exposed cortex to prevent drying. A high-resolution photo was taken of the cortical surface, to be used later to reference electrode penetration sites.

### Auditory stimuli

Stimuli were generated using Tucker-Davis Technologies (TDT) System 3 hardware and presented at a rate of 223214.2857 samples/s through a free-field speaker, which was fed by a PA5 programmable attenuator and SA1 stereo amplifier module. Experimental stimuli consisted of pure tones (30 frequencies log-spaced 4–80 kHz, seven sound intensities in 10 dB steps from 5 to 65 dB SPL, 60 ms duration) and USVs (selected from a library of pup USVs routinely used by our laboratory). USVs were high-pass filtered in MATLAB (25 kHz corner, 8-order Butterworth filter), denoised, and Hilbert transformed to extract the frequency and amplitude envelopes. These envelopes were used to resynthesize a clean version of each USV against a silent background. These stimuli were convolved with a 0.5 ms cos^2^ onset/offset function, and scaled to a maximum amplitude of 65 dB SPL. Stimuli were presented with a 600 ms interstimulus interval, and repeated 15 times each in pseudorandom order through a free-field speaker (EMIT high-energy speaker; Infinity Systems) positioned 11 cm lateral to the right ear.

### Multiunit electrophysiology

**Figure 1.**
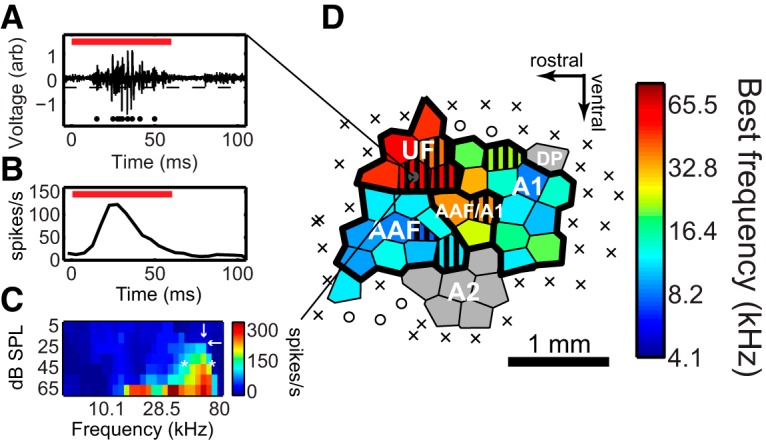
Multiunit electrophysiology methods. ***A***, Voltage trace of a sound-evoked multiunit response. The red bar indicates tone playback (60 ms), and the dashed line represents the user-defined spiking threshold. ***B***, Sample peristimulus time histogram of sound-evoked multiunit spiking responses. The red bar indicates tone playback. ***C***, Frequency response area for a sample multiunit. Spiking responses are indicated with hot colors representing a greater evoked response; responses at seven sound amplitudes (5–65 dB SPL, in 10 dB steps) and 30 frequencies (4–80 kHz log spaced) are shown. Downward white arrow indicates BF; leftward white arrow indicates threshold; white asterisks indicate lower and upper boundaries of the bandwidth at 20 dB above threshold. ***D***, A sample Voronoi tessellation representing the tonotopic map of an animal. Hot colors represent higher BFs. Auditory regions are labeled according to region delineations defined in the study by Stiebler et al. (1997). Core AC areas (UF, AAF, AAF/A1, and A1) are outlined in a thick black line. Non-core auditory areas (A2, DP) are in gray. Hashed sites respond to USVs. X indicates areas that are not tone responsive.

Unit recordings were taken across the left AC using a 4 MΩ 3 × 1 tungsten matrix microelectrode (FHC) with 305 μm interelectrode spacing. The electrode was driven into thalamorecipient layer 4 (∼400 µm) by a micromanipulator with hydraulic microdrive (FHC). Electrode penetration sites were labeled on the high-resolution photo of the AC by cross-referencing local vascular landmarks between the photograph and the live microscope image. After a short adaptation period, recording and stimulus playback were initiated. Stimulus playback and data acquisition were coordinated by TDT System 3 hardware controlled by the BrainWare application using modules programmed in the RPvdsEx environment. Electrophysiological signals were sampled at 24 kilosamples/s, passed through an RA16AC high-impedance headstage, amplified with a RA16PA Medusa preamplifier (TDT), and filtered at >300 Hz and <6 kHz, with a notch at 60 Hz. Spikes were detected using a negative threshold set by the experimenter (Fig. 1*A*). In setting the threshold, effort was made to isolate clearly defined spikes and exclude the increased “hash” that often accompanies evoked firing in multiunit recordings. While these criteria were qualitative, *post hoc* analysis of a subset of recordings indicates that the threshold was set such that it exceeded the average root mean square of the signal during spontaneous activity by a factor of 5, on average.

### Data analysis

Off-line, peristimulus time histograms were created by pooling spikes evoked by all trials (i.e., tones of all frequency/intensity combinations) from a given recording (Fig. 1*B*). A blinded experimenter manually determined the temporal boundaries of the excitatory phase of the evoked response. Frequency response areas (FRAs) were constructed for each multiunit by plotting the magnitude of the response (in spikes per second), integrated over this time window, against the frequency and amplitude of the stimulus (Fig. 1*C*). A blinded observer referenced the FRA of each unit to manually determine its threshold. BFs were extracted in MATLAB (MathWorks) and defined as the frequency that generated the highest average spike rate over all the intensities equal to or greater than the threshold intensity. The width of pure tone frequency tuning was characterized by a Quality factor (Q20), derived by dividing the BF by the bandwidth at 20 dB above threshold, where bandwidth approximates the FRA tuning width at the half-maximum firing rate. Hence, wider frequency tuning bandwidths correspond to lower Q20 values.

In all experiments, efforts were made to surround auditory-responsive sites with a perimeter of nonresponsive sites, ensuring that the complete spatial extent of AC was captured. In these cases, BF maps were constructed by performing Voronoi tessellations on all recording sites for a given animal (Fig. 1*D*). These maps were used to provide a qualitative impression of AC organization and to enable area-based measurements of characteristics of interest (e.g., area of cortex responsive to a given frequency). To construct a BF map, the *x*- and *y*-coordinates of all recording sites were first extracted from the high-resolution AC photo using open-source software (DataThief), and the voronoin function in MATLAB was used to return their estimated boundaries. Area-based measurements were calculated by summing the areas of the polygons associated with recording sites. A fraction of the recording sites corresponded to polygons with infinite or spuriously large areas because for technical reasons we were unable to “enclose” that area with a perimeter of nonauditory sites. To ensure that these areas did not bias our analyses, we reassigned the 5% of sites with the largest areas to the median area measurement.

Recording sites were assigned to one of the five subregions of the mouse AC using classification criteria outlined in the study by Stiebler et al. (1997). Briefly, subregion designations were based on the following attributes of the recording site: (1) spatial position within AC; (2) BF; (3) response latency; (4) tuning bandwidth; (5) responsiveness to USVs (if USVs were presented); and (6) the tendency to habituate to repeated presentations of a stimulus. Core auditory subregions [A1, anterior auditory field (AAF), and ultrasonic field (UF)] can be characterized by the short response latencies (<15 ms) of their neurons; sharp tuning bandwidths; and reliable, nonfatiguing onset responses to repeated presentations of a stimulus (Joachimsthaler et al., 2014). Noncore subregions [secondary auditory (A2) and dorsal posterior field (DP)] have longer response latencies (>15 ms), wide or multipeaked tuning curves, and a tendency to habituate to repeated presentations of a stimulus (Stiebler et al., 1997). These sites also tend to be more responsive to frequency-modulated sounds (like USVs), relative to pure tones with flat frequency trajectories. These criteria helped to classify recording sites as core or noncore; frequency tuning and spatial position within the map helped to further determine the precise subregion. A core recording site with an ultrasonic BF (>40 kHz) was determined to be part of the UF so long as it was located rostrodorsally. Core sites with BFs <40 kHz were assigned to either AAF or A1, depending on their position relative to the high-frequency band that separates AAF from A1. Specifically, sites rostral or caudal to that band were designated AAF or A1 sites, respectively. Noncore sites positioned caudodorsally relative to the core AC were assigned to area DP, whereas those appearing ventrally were assigned to area A2. Subregions were assumed to be spatially contiguous. When these criteria conflicted in a way that precluded an obvious subregion assignment (e.g., the spatial position of a site was consistent with an assignment to subregion UF, but its BF was <40 kHz), the subregion of the site was designated “unknown.” Additionally, sites located in the high-frequency band that separates A1 and AAF were assigned to a transitional region “AAF/A1.” These sites, like those in A1, AAF, and UF, were considered as part of the core AC.

In some cases, complete maps could not be obtained from experimental animals due to experimental difficulties. These partial maps (*n* = 9: 3 naive, 4 P3–P4 dams, 1 P9–P10 dam, 1 postwean dam) were not included in area-based analyses (e.g., proportionate area of AC tuned to a given frequency range). However, multiunit recordings from these partial maps were included in non-area-based analyses (e.g., average response properties associated with particular subregions) if enough of the AC had been mapped to permit confident assignment of units to AC subregions.

To compare average responses in a standardized fashion, the response strength to USV playback was calculated by taking the firing rate over a uniform 100 ms poststimulus time window. Units were then judged responsive or nonresponsive to each USV exemplar by an automated MATLAB algorithm that compared the distribution of these firing rates to the distribution of spontaneous firing rates (calculated over a 100 ms window beginning 200 ms prior to stimulus onset) using a two-sample *t* test. USVs were judged to have elicited a response if the *t* test returned a *p* value <0.001 (to account for a large number of comparisons). These criteria reasonably approximated human judgment of stimulus-driven responsiveness.

### Statistical analysis

Lilliefors tests were conducted on datasets to confirm that they were normally distributed. Satisfying this, data were then analyzed with a one-way ANOVA to compare means across naive animals and the three maternal groups. When analyses yielded a significant effect (*p* < 0.01), Tukey’s HSD test was used to correct for multiple pairwise, *post hoc* comparisons (*p < 0.01 taken as significant in figures; #*p* < 0.05 taken as marginally significant in figures). When the ANOVA was only marginally significant (*p* < 0.05), *post hoc* tests are reported in the text only.

## Results

This study aimed to determine whether tonotopic map plasticity occurs in maternal animals following early experience with pups and their USVs. Recordings were performed in the thalamorecipient layer of the left AC, which is believed to play a dominant role in communication sound processing across species (Cutting, 1974; Petersen et al., 1978; Ehret, 1987; Marlin et al., 2015). CBA/CaJ dams whose pups were either P3–P4 or P9–P10, bracketing the time point when pup USV emission rates generally peak (Haack et al., 1983), were compared to naive virgins and postweaning dams. In total, 1,484 multiunit recordings were taken from 40 anesthetized female mice spanning all four animal groups, and these were used to construct complete maps of AC for 31 animals (naive: 416 multiunits total/326 in complete maps; P3–P4 dam: 384/278; P9–P10 dam: 336/316; post-weaning dam: 348/322). Table 1 reports the average absolute and proportional sizes of core AC fields, following the original nomenclature from the study by Stiebler et al. (1997).

**Table 1:** Average size of AC fields in the CBA/CaJ mouse, expressed in absolute mm^2^ and as a proportion of total AC area

Region	Absolute area (mm^2^)	Proportional area (%)
All AC	3.74 ± 0.54	100
UF	0.45 ± 0.14	12.1 ± 3.9
AAF/A1	0.22 ± 0.11	5.9 ± 2.8
AAF	0.92 ± 0.29	24.8 ± 7.5
A1	1.17 ± 0.24	31.3 ± 5.3
A2	0.62 ± 0.26	16.2 ± 6.1
DP	0.22 ± 0.13	5.8 ± 3.5

Values are reported as the mean ± SD.

**Figure 2.**
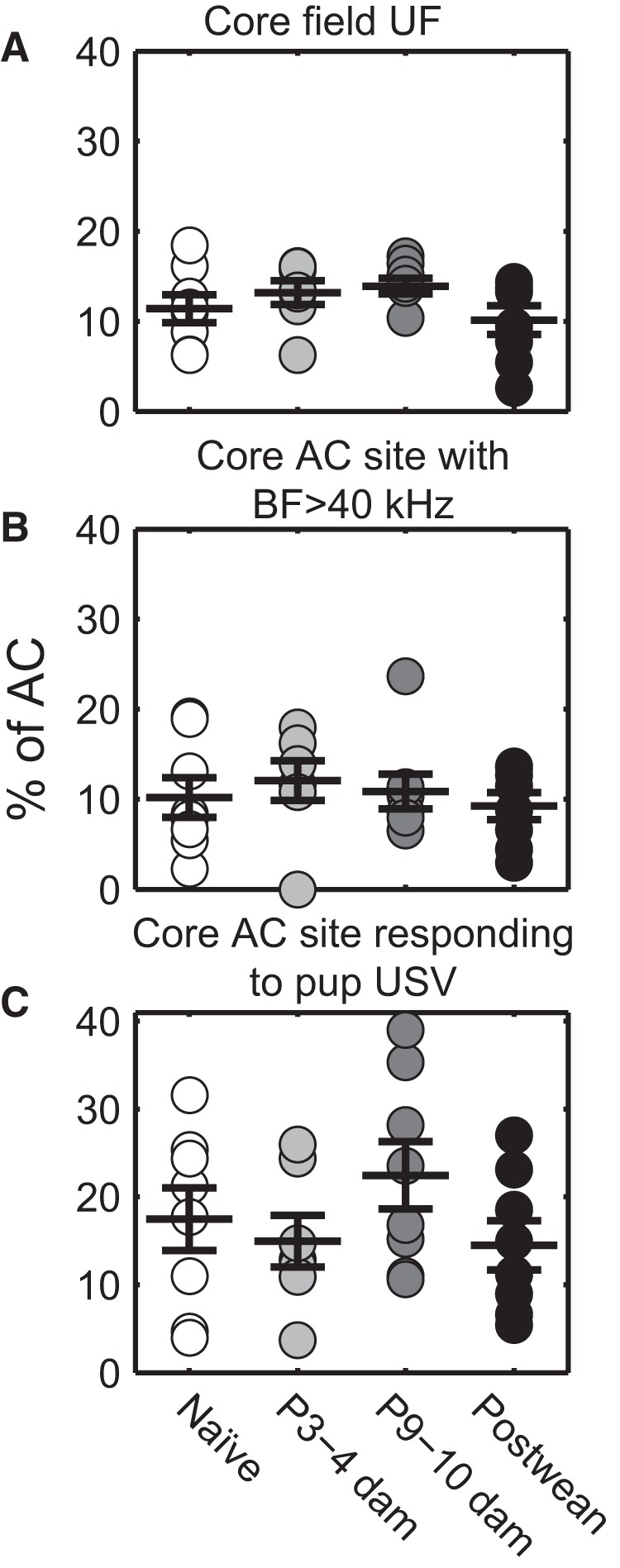
Map representation over maternal experience: naive (*n* = 8), P3–P4 (*n* = 7), P9–P10 (*n* = 8), Postweaning (*n* = 8). ***A***, No expansion of the proportionate area of UF across maternal experience, one-way ANOVA: *F*_(3,27)_ = 1.59, *p* = 0.22. ***B***, No expansion of areas tuned to ultrasound frequencies across maternal experience, BF >40 kHz, one-way ANOVA: *F*_(3,27)_ = 0.76, *p* = 0.52. ***C***, No expansion of proportionate areas responsive to pup USVs across maternal experience, one-way ANOVA: *F*_(3,27)_ = 1.21, *p* = 0.33. Filled circles represent individual mice. Lines and error bars in all panels indicate the mean ± standard error of the mean (SEM).

Since sites with BFs in the ultrasound range, where pup calls reside, are generally found in the core field UF, which is functionally (rather than anatomically) defined, we first asked whether UF may increase in size over the course of motherhood. The proportionate size of UF as part of the entire AC, however, did not differ with maternal experience (Fig. 2*A*; one-way ANOVA: *F*_(3,27)_ = 1.59, *p* = 0.22). Further, there was no overall difference in absolute AC size over the course of motherhood (one-way ANOVA: *F*_(3,27)_ = 0.39, *p* = 0.76), nor were there any apparent changes in proportionate size for any other AC field (one-way ANOVAs: AAF/A1: *F*_(3,27)_ = 1.82, *p* = 0.17; AAF: *F*_(3,27)_ = 1.04, *p* = 0.39; A1: *F*_(3,27)_ = 0.23, *p* = 0.88; A2: *F*_(3,27)_ = 0.98, *p* = 0.42; DP: *F*_(3,27)_ = 0.1, *p* = 0.96). Thus, experience with pup USVs did not create significant relative areal expansion or contraction in any AC subfield over the course of motherhood.

We next investigated the possibility that the core AC was reorganized at the map level to over-represent behaviorally relevant ultrasonic BFs in maternal mice, but that the retuned core sites were outside a spatially contiguous UF (see Materials and Methods). We therefore assessed the proportionate area of core AC sites with BF >40 kHz, as a fraction of the whole AC. This analysis, too, provided no evidence for map plasticity (Fig. 2*B*; one-way ANOVA: *F*_(3,27)_ = 0.76, *p* = 0.52). Further, the BF distributions of each core AC subregion did not differ throughout motherhood (data not shown). Perhaps instead of expansion of areas tuned to ultrasound pure tones, the amount of core AC that responds specifically to pup USVs (Fig. 1*D*, hashed areas) increases over motherhood. Since a site does not need to have an ultrasonic BF to respond to USVs (Portfors et al., 2009; Shepard et al., 2015b), we next assessed the area of core AC that simply responds to any of the pup USVs we presented, as a fraction of the whole AC. This analysis also provided no evidence for map plasticity, as the pup USV-responsive area does not change across motherhood (Fig. 2*C*; one-way ANOVA: *F*_(3,27)_ = 1.21, *p* = 0.33).

Together, these analyses fail to support the hypothesis that BF map plasticity occurs transiently during motherhood for frequencies in the ultrasound range following experience with pup USVs. Instead, even though there is interindividual variability, the multiunit-based map of the core AC appears relatively stable throughout motherhood, at least with respect to its subfield organization and the spatial organization of its ultrasonic frequency responses.

Although BF distributions do not apparently change in the maternal AC, systematic changes within the receptive field of a unit could still alter how units collectively respond to ultrasound frequencies, without shifting BFs. Indeed, in core AC, ultrasound-evoked inhibition of single units tuned to low frequencies (<40 kHz) is stronger in mothers than in pup-naive females, while the strength of inhibition in neurons with high BFs (>40 kHz) is comparable between groups (Galindo-Leon et al., 2009; Lin et al., 2013). Ultrasound-evoked overall excitation of single units also showed no such modulation by maternal status (although distinct subgroups of neurons do; Shepard et al., 2015b). This observation led to the hypothesis that, upon ultrasound presentation, net population activity (excited–inhibited) in low-frequency-tuned regions of core AC (i.e., A1 and AAF), but not in area UF, would be suppressed to a greater degree in mothers compared with naive females. The result would produce a greater contrast in activity between high- and low-frequency-tuned regions of core AC in mothers, possibly facilitating better detection of ultrasound frequencies at downstream sites.

**Figure 3.**
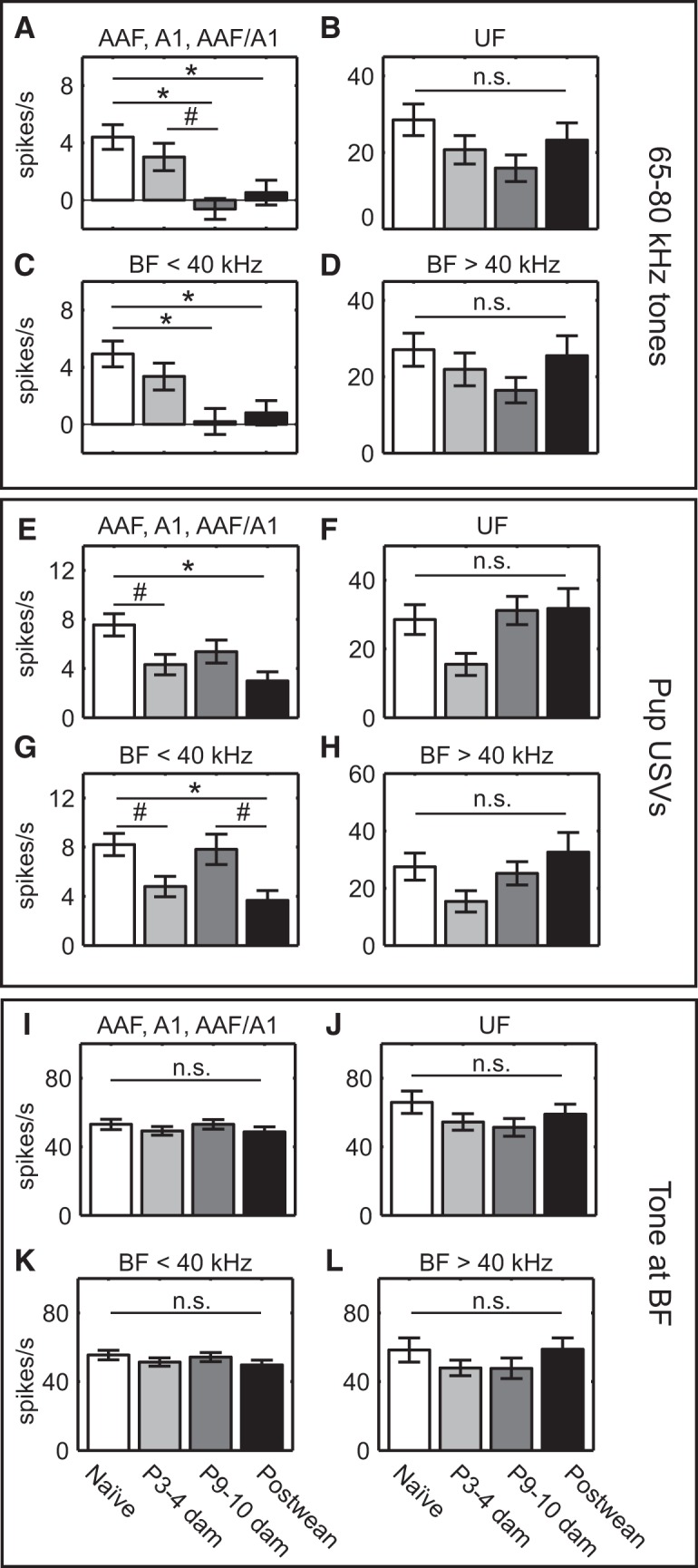
Animals with more maternal experience show enhanced suppression of responses to ultrasound stimuli in low-frequency-tuned areas. ***A–D***, Across maternal experience, evoked firing rates in response to playback of 65-80 kHz pure tones in AAF, A1, AAF/A1 (***A***); UF (***B***); areas with BF <40 kHz (***C***); and areas with BF >40 kHz (***D***). Low-frequency-tuned areas and AAF/A1 show enhanced suppression at P9–P10 and postweaning time points. ***E–H***, Evoked firing rates in response to playback of pup USVs in AAF, A1, AAF/A1 (***E***); UF (***F***); areas with BF <40 kHz (***G***); and areas with BF >40 kHz (***H***). Low-frequency-tuned areas show enhanced suppression in response to pup USVs at the postweaning time point. ***I–L***, Evoked firing rates in response to playback of a pure tone at the BF of the region in AAF, A1, AAF/A1 (***I***); UF (***J***); areas with BF <40 kHz (***K***); and areas with BF >40 kHz (***L***). No difference in firing rates to the BF of a site is seen across maternal experience. Error bars in all panels indicate the SEM. #*p* < 0.05, *p < 0.01, *post hoc* Tukey’s HSD. n.s, Not significant.

To test this hypothesis, we averaged the firing rate responses to a subset of ultrasound tones (65–80 kHz, 55–65 dB SPL) over a 100 ms time window beginning at stimulus onset. This sound-level range was chosen to match the 65 dB SPL amplitude used to present USVs, which corresponds to an ethologically relevant sound pressure when a dam is ∼20 cm away from a pup (Haack et al., 1983). For each multiunit, responses were normalized by subtracting the spontaneous rate. Averaging together the normalized firing rates of all AAF, A1, and AAF/A1 units per group revealed a progressive suppression of ultrasound responses (Fig. 3*A*; one-way ANOVA: *F*_(3,944)_ = 6.76, *p* = 0.0002). Ultrasound-evoked firing rates in these “lateral-band” fields were near zero in postwean dams and P9–P10 dams; the evoked firing rates of both of these groups were significantly lower than those of naive animals. Firing rates observed in P3–P4 dams were not lower than those of naive animals but were marginally greater than those of P9–P10 dams. Consistent with our hypothesis, no such suppression occurred for UF sites (Fig. 3*B*; one-way ANOVA: *F*_(3,186)_ = 1.9, *p* = 0.13). Similar results were observed when core units were grouped by BF rather than subfield. Firing rate responses of core multiunits with BFs <40 kHz in P9–P10 and postwean dams were significantly lower than those observed in naive mice (Fig. 3*C*; one-way ANOVA: *F*_(3,973)_ = 5.86, *p* = 0.0006), but no significant suppression was observed among core units with BFs >40 kHz (Fig. 3*D*; one-way ANOVA: *F*_(3,157)_ = 1.18, *p* = 0.32).

To further validate this hypothesis, we next examined firing rates evoked by the most prototypical pup USV exemplar (USV 1). Again, the suppression of responses to this USV was observed in AAF, A1, and AAF/A1 multiunits in mothers, relative to naive animals (Fig. 3*E*; one-way ANOVA: *F*_(3,914)_ = 5.16, *p* = 0.002). As in the ultrasound tone analysis, postweaning dams exhibited a significant suppression of the evoked response to USVs relative to naive mice. Marginally significant suppression relative to naive mice was also observed in P3–P4 dams, though not in P9–P10 dams. Among UF multiunits, modulation of the USV response magnitude across animal groups did not reach our level of significance (Fig. 3*F*; one-way ANOVA: *F*_(3,187)_ = 3.25, *p* = 0.02), although the marginally significant *F*-statistic was followed by only a marginally significant (*post hoc*) difference between P3–P4 and P9–P10 dams (*p* = 0.04). These same trends were apparent when core units were grouped by BFs >40 kHz (Fig. 3*G*; one-way ANOVA: *F*_(3,955)_ = 5.51, *p* = 0.0009) and <40 kHz (Fig. 3*H*; one-way ANOVA: *F*_(3,155)_ = 2.21, *p* = 0.089).

As an important control for nonspecific modulation of evoked firing over the course of motherhood, we also calculated for each multiunit its average firing rate response to its BF tone at 55–65 dB SPL. In no case was there a significant modulation of BF responses, whether we analyzed AAF, A1, and AAF/A1 multiunits as a group (Fig. 3*I*; one-way ANOVA: *F*_(3,825)_ = 0.7, *p* = 0.55), UF multiunits (Fig. 3*J*; one-way ANOVA: *F*_(3,189)_ = 1.34, *p* = 0.26), or core units with BF <40 kHz (Fig. 3*K*; one-way ANOVA: *F*_(3,965)_ = 0.99, *p* = 0.39) or >40 kHz (Fig. 3*L*; one-way ANOVA: *F*_(3,156)_ = 1.02, *p* = 0.39).

**Figure 4.**
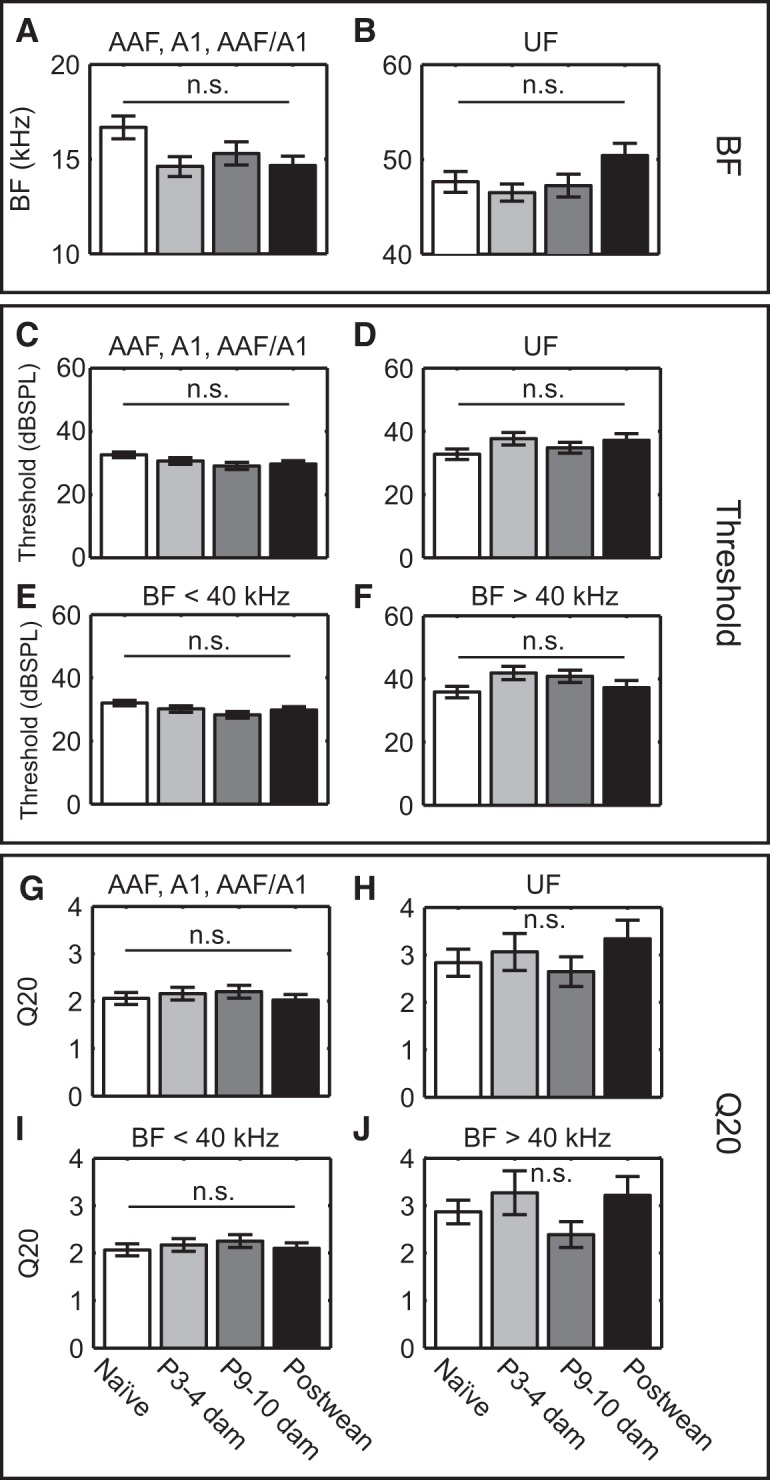
Increased lateral-band suppression cannot be explained by changes in multiunit tuning across maternal experience. ***A***, ***B***, Average BF of multiunits in AAF, A1, and AAF/A1 (***A***); and UF did not vary significantly over motherhood (***B***). ***C–F***, Pure tone thresholds for multiunits in AAF, A1, AAF/A1 (***C***); UF (***D***); core areas with BF <40 kHz (***E***); and core areas areas with BF >40 kHz (***F***) were not significantly modulated by motherhood. Tuning bandwidths as measured by Q20 values also did not differ significantly in AAF, A1, AAF/A1 (***G***); UF (***H***); areas with BF <40 kHz (***I***), and areas with BF >40 kHz (***J***). Error bars in all panels indicate the SEM. n.s, Not significant.

As a test of whether changes in frequency tuning could explain the reduced USV response, we compared the average BF, tuning curve thresholds, and Q20 values across motherhood. Consistent with the mapping results, animal group differences in average BFs for multiunits in AAF, A1, and AAF/A1 (Fig. 4*A*; one-way ANOVA: *F*_(3,944)_ = 3.17, *p* = 0.02) and UF (Fig. 4*B*; one-way ANOVA: *F*_(3,186)_ = 2.02, *p* = 0.11) did not reach significance, although the marginally significant *F*-statistic for the former was followed by only a marginally significant (*post hoc*) difference between naive mice and P3–P4 dams (*p* = 0.03). The same was true for the characteristic frequency at threshold (data not shown; AAF, A1, and AAF/A1 one-way ANOVA: *F*_(3,944)_ = 2.62, *p* = 0.05; UF one-way ANOVA: *F*_(3,186)_ = 1.04, *p* = 0.38). Similarly, thresholds did not change significantly in either AAF, A1, and AAF/A1 (Fig. 4*C*; one-way ANOVA: *F*_(3,944)_ = 2.31, *p* = 0.08) or UF (Fig. 4*D*; one-way ANOVA: *F*_(3,186)_ = 1.48, *p* = 0.22). The same was true when segregating core units by BF <40 kHz (Fig. 4*E*; one-way ANOVA: *F*_(3,973)_ = 2.37, *p* = 0.07) or >40 kHz (Fig. 4*F*; one-way ANOVA: *F*_(3,157)_ = 2.17, *p* = 0.09). Q20 values were also unchanged across motherhood, whether we analyzed multiunits in AAF, A1, and AAF/A1 (Fig. 4*G*; one-way ANOVA: *F*_(3,758)_ = 0.39, *p* = 0.76), UF (Fig. 4*H*; one-way ANOVA: *F*_(3,152)_ = 0.69, *p* = 0.56), or core units with BF <40 kHz (Fig. 4*I*; one-way ANOVA: *F*_(3,796)_ = 0.4, *p* = 0.75) or >40 kHz (Fig. 4*J*; one-way ANOVA: *F*_(3,121)_ = 1.22, *p* = 0.30). Not surprisingly, therefore, the evoked firing rate response to 65–80 kHz tones at 10–20 dB above the threshold of each unit was also not significantly changed (data not shown), irrespective of whether the unit was in AAF, A1, and AAF/A1 (one-way ANOVA: *F*_(3,805)_ = 0.31, *p* = 0.82), and UF (one-way ANOVA: *F*_(3,187)_ = 1.05, *p* = 0.37), or had a BF <40 kHz (one-way ANOVA: *F*_(3,943)_ = 0.57, *p* = 0.64) or >40 kHz (one-way ANOVA: *F*_(3,152)_ = 0.77, *p* = 0.51).

Together, these results demonstrate that despite a lack of change in pure-tone tuning widths, ultrasound-evoked activity for ethologically relevant moderate sound levels is specifically suppressed among low-frequency-tuned units in maternal mice, while the response strength in high-frequency-tuned areas is not changed by maternal experience. This creates greater contrast between the low- and high-frequency-tuned neural populations in core AC when stimulated by USVs.

## Discussion

Tonotopic map area expansion is a frequently reported manifestation of adult cortical plasticity for sound frequencies that acquire behavioral relevance, at least transiently (Reed et al., 2011) if not more permanently (Weinberger et al., 1993). However, we failed to observe it for pup-associated ultrasonic frequencies in maternal animals having very recent experience with their young, despite motherhood inducing a behavioral preference for pup USVs and ultrasound tones (Ehret et al., 1987; Uematsu et al., 2007; Lin et al., 2013). Instead, USVs evoke progressively stronger lateral-band suppression as they gain behavioral relevance, validating and extending a previous conclusion using a completely different experimental design and analysis (Galindo-Leon et al., 2009; Lin et al., 2013; Elyada and Mizrahi, 2015). Hence, as a vocal category becomes behaviorally meaningful, experience-dependent core AC reorganization does not produce a spatial over-representation of the spectral content of the vocalization but enhances the population-level contrast between call-excited neural activity evoked in areas tuned to versus away from that spectral content.

Neuronal plasticity that enhances excitatory responses to pup USV features is observed both during motherhood (Cohen et al., 2011; Cohen and Mizrahi, 2015) and afterward (Liu et al., 2006; Liu and Schreiner, 2007; Marlin et al., 2015; Shepard et al., 2015b), but it had been an open question whether such changes in an ethological learning context are accompanied by spatial map expansion. Shepard et al. (2015b) reported that postweaning dams do not show relative expansion for ultrasonic sounds within core AC, but it was unclear whether transient expansion could arise to support learning (Kilgard, 2012). Cohen et al. (2011) initially reported a relative increase in USV-responsive single units in the lower BF region of A1 in P5 lactating dams compared with naive mice, feeding the expectation of concomitant areal plasticity. This increase diminished in postweaning dams, although, like Galindo-Leon et al. (2009), it remained significantly higher than that in naive mice. However, the follow-up study by Rothschild et al. (2013) did not see the increase in cortical layer 2/3 of lactating dams, perhaps because it is confined to deeper layers. Without uniform and complete sampling across the entire AC of individual animals though, increases in single-unit responsiveness percentages do not necessarily imply increases in the cortical area engaged by the sound. While our negative results on spatial representational expansion in the thalamorecipient layer of cortex are based on a lower spatial sampling density than that used in other mouse studies (Guo et al., 2012; Kim et al., 2013), we note that the same methods were sufficient to reveal map expansion in a developmental sound exposure paradigm (Shepard et al., 2015a). Perhaps brief expansion occurs at some other maternal time point outside of those used here to bracket the peak in pup calling rates. However, this seems unlikely since conditioning studies suggest that expansion should remain for at least ∼20 d (Weinberger et al., 1993; Reed et al., 2011) after the start of sound training (i.e., parturition here); expansion arising after P10 would then have been visible in our postweaning mice.

Lack of map expansion in core AC is consistent with this topographical form of AC plasticity being sensitive to the details of how the meaning of a sound is learned (Bieszczad and Weinberger, 2010c), which can differ between laboratory conditioning and ethologically relevant experiences. For example, laboratory conditioning traditionally relies on nonsocial reinforcers (e.g., food as reward or footshock as punishment) to render a sound salient, whereas communication sounds gain behavioral relevance through social interactions with conspecifics (e.g., social reward of pups; Hauser and Gandelman, 1985; Fleming et al., 1994; Ferris et al., 2005). Social reinforcers recruit distinct hormonal mechanisms for plasticity (Remage-Healey et al., 2010; Banerjee and Liu, 2013; Marlin et al., 2015), potentially affecting the neural correlates of learning. Indeed, even among nonsocial reinforcers, different receptive field effects are observed when appetitive instead of aversive operant conditioning is used (David et al., 2012). Much remains to be understood about how learning-related neuromodulatory systems are engaged to dynamically (Marlin et al., 2015; McGinley et al., 2015) or more persistently (Kilgard and Merzenich, 1998; Bao et al., 2001; Ji and Suga, 2003) alter AC responses to sounds, but the sensitivity to learning context motivates pursuing ethological paradigms to reveal intrinsic mechanisms of communication sound learning (Bennur et al., 2013).

How might we functionally explain the absence of map plasticity in this ethological context, despite plasticity on a finer single and multiunit scale (Liu and Schreiner, 2007; Galindo-Leon et al., 2009; Cohen et al., 2011; Marlin et al., 2015)? Plasticity in AC, whether at the level of maps or not, is generally thought to be a key mechanism for perceptual learning (Polley et al., 2006; Froemke and Schreiner, 2015). Map plasticity seems to be more robust when the perceptual cue being learned depends on the onset of the sound (Bieszczad and Weinberger, 2010c), which in most laboratory conditioning paradigms is a pure tone of a single specific frequency. Lack of map plasticity might therefore indicate that the onset frequency of a USV is not a learned feature for recognizing pup calls. In fact, since pup USVs share a range of ultrasonic onset frequencies similar to those of another USV category emitted by adult males (Liu et al., 2003), this cue alone may be less informative than perceiving how the ongoing frequency of the vocalization is modulated, a feature for which plasticity at the single-unit level is observed (Shepard et al., 2015b). Alternatively, map expansion observed for nonsocial conditioning paradigms may derive from the over-reliance on a single pure tone frequency to convey meaning, which does not occur for natural vocal categories with intrinsic acoustic variability (Liu et al., 2003). That spectrotemporal variability may distribute plasticity across the tonotopic axis, thereby diluting the effect of enhancing the representation of any one frequency.

AC plasticity may also play a more direct role in mapping stimulus cues to behavioral responses (Znamenskiy and Zador, 2013). In operant training, not only is the meaning of a sound cue being learned, but often also a new motor response (e.g., bar press) to that cue. Transient AC map expansion during learning may enable the exploration of more optimal sensorimotor pathways for stimuli to transduce appropriate behavioral responses (Kilgard, 2012). In the case of an ethological behavior whose underlying motor response is instinctive (i.e., retrieval occurs spontaneously when naive mice encounter pups in their home cage; Numan and Insel, 2003), perhaps such neural exploration is not necessary. Representations of USVs along the auditory pathway may already be innately expanded (Portfors et al., 2009; Issa et al., 2014; Garcia-Lazaro et al., 2015), and/or maternal hormones may simply unlock a latent auditory circuit tuned to respond to pup USVs (Bargmann, 2012; Wu et al., 2014). Whether the lack of map plasticity reflects the “specialized” nature of an evolved USV communication system could then be investigated by training a synthetic sound category as an appetitive cue to trigger the search and retrieval of socially rewarding pups, which would help to facilitate comparisons between studies of auditory memory that make use of laboratory versus ethological learning paradigms.

Lack of map expansion notwithstanding, the auditory cortices of maternal animals did acquire a large-scale trace of their experience with pup USVs: in core areas tuned to low, but not high, frequencies, the magnitude of the neural response to moderate sound level pup USVs was dampened toward spontaneous levels in dams with at least 9–10 d of pup care experience. Because this effect was not accompanied by a significant change in the magnitude of the auditory response in high-frequency-tuned areas, we can surmise that the activity of the maternal core auditory cortex as a whole exhibits sharper contrast between high- and low-frequency regions upon presentation of behaviorally relevant ultrasound frequencies. This is achieved not by “turning up” the overall average magnitude of the auditory response in all neurons tuned to ultrasonic frequencies (though gain increases are seen in specific subsets of pyramidal neurons; Shepard et al., 2015b), but instead, by suppressing the “background chatter” of neurons residing in the so-called lateral band, which flanks the ultrasonic frequency range of interest. Interestingly, such lateral band suppression was apparent for a moderate absolute sound level, rather than when averaging across relative levels just above the threshold of a unit. This suggests that neural coding strategies at disparate absolute sound levels can be different, as previously reported for temporal coding at moderate versus low sound levels (Nagel and Doupe, 2006).

Our findings confirm and extend earlier work that found evidence of lateral band suppression in single auditory neurons upon ∼65 dB SPL USV presentation following natural maternal experience (Galindo-Leon et al., 2009; Lin et al., 2013). In the previous studies, while the proportion of USV-inhibited single units did not differ between pup-naive animals and experienced mothers, the depth and duration of that inhibition was longer in maternal single units, but only when they were tuned to frequencies below the ultrasound range. There was no concomitant change in USV-inhibited neurons tuned to ultrasound frequencies, or in average USV-evoked excitation. In summing USV-evoked single-unit excitation and inhibition to predict the population response of core auditory cortical neurons, the combination in lateral band regions of deeper inhibition and stable excitation in mothers should then lead to a suppression of the neural activity of that area upon USV presentation. Our new data now confirm this by using multiunits, suggesting that when learning natural communication sounds in adulthood, the auditory cortex uses a strategy to reduce the relative firing in, instead of the amount of territory of, regions attuned away from the frequency of the behaviorally relevant sound category. Enhanced contrast could facilitate detection of the behaviorally relevant ultrasonic stimulus in a downstream brain region that receives aggregated input from high- and low-frequency-tuned regions of the core auditory cortex. Indeed, there is evidence that just such a suppressive effect occurs on shorter time scales to improve sound detection: when ferrets were trained to detect pure tones in a high-noise environment, the activity of single auditory cortical neurons with BFs far from the frequency of the target tone underwent rapid suppression during the task (Atiani et al., 2009). Our observations here may reflect the same functional plasticity in a consolidated form.

That this suppressive effect stems from changes in inhibition gains credence from several recent studies in which inhibition or disinhibition are primary drivers of experience-dependent plasticity in AC. Letzkus et al. (2011) and Kato et al. (2015) each showed enhanced excitation in layer 2/3 pyramidal neurons during and after behavioral conditioning, apparently as a result of disinhibition by local inhibitory interneurons. Others have even found direct evidence of inhibitory AC plasticity in the maternal context itself. Marlin et al. (2015) showed that playing pup USVs to pup-naive females in the presence of oxytocin, a potent neuropeptide involved in maternal behavior and pair bonding, promoted lasting USV-evoked excitation among neurons in the left core AC. This effect was precipitated by rapid disinhibition; whole-cell recordings taken during USV-oxytocin pairing revealed a decrease in inhibition that preceded the enhancement of excitation by minutes. Endogenously in mothers, such disinhibition may perhaps be triggered by pup odors, as recent work (Cohen and Mizrahi, 2015) found that exposing maternal mice to pup odors disinhibited, and subsequently facilitated, excitatory responses to USVs in layer 2/3 pyramidal neurons. Importantly here, that study also showed that motherhood shifts the odor-independent BFs of parvalbumin-expressing inhibitory neurons, but not pyramidal neurons, higher by ∼1 octave into a region where they would be activated by USVs. This finding not only suggests that greater USV-evoked inhibition in mothers could underlie the lateral-band suppression we confirmed here, but also indicates that inhibitory plasticity likely arises *de novo* in the cortex rather than in subcortical stations. In summary, our work provides new validation for the growing consensus that, while sensory cortical plasticity is often reported as a change in the excitatory response to a relevant stimulus, alterations in inhibitory physiology are often underlying.

## Synthesis

Reviewing Editor: Leonard Maler, University of Ottawa Decisions are customarily a result of the Reviewing Editor and the peer reviewers coming together and discussing their recommendations until a consensus is reached. When revisions are invited, a fact-based synthesis statement explaining their decision and outlining what is needed to prepare a revision will be listed below. The following reviewer(s) agreed to reveal their identity: Robert Froemke.

Both reviewers now agree that this Ms makes a worthwhile contribution to the literature in this field and is suitable for publication in eNeuro.

Your revision should address a remaining concern of the second reviewer.

Please make sure to clearly note the changes you have made as they will only be reviewed by the Editor

Reviewer #2

See my main comment in the review: if the p value reported in the text is a typo, the statistical analysis is correct and the typo simply needs to be fixed. If the p value currently reported is the correct p value, then the authors simply need to perform an additional post-hoc test.

Comments to the Authors

The authors have adequately addressed my previous concerns. Inclusion of threshold and tuning analyses has strengthened the interpretation of their findings. This is a solid, well-written paper. I have only a single final comment:

Major:

Page 15, lines 7-8: The authors claim that the group differences in average BFs for multi-units in AF,A1 and AAF/A1 do not differ significantly, yet they report p = 0.02. Is this a typo? If not, it is unclear why in this specific instance, the authors have chosen to represent this finding as insignifcant. In other parts of the paper (e.g. Fig 3A), p = 0.05 is accepted as at least "marginally significant." I suggest that the text should be modified to more accurately reflect this finding, and post-hoc tests should be performed to determine which groups differ from one another. Also, the y-axis on Fig 4A should be rescaled so that the differences between groups can be seen more clearly. If I look very carefully, it appears that the BF of multi-units in AAF,A1 and AAF/A1 is lower in postweaning dams compared to naive dams. How do the authors interpret this finding in the context of their other results?
